# Impact of technology-assisted versus manual sterile compounding on safety and efficiency in a Canadian community hospital

**DOI:** 10.1093/ajhp/zxac167

**Published:** 2022-06-15

**Authors:** Mark Fan, Danny Yang, Becky Ng, Jocelyn Jackson, Katherine Bouris, Sharon Eng, Edith Rolko, Patricia Trbovich

**Affiliations:** Centre for Research and Innovation, North York General Hospital, Toronto, ON, Canada; Centre for Research and Innovation, North York General Hospital, Toronto, ON, Canada; Centre for Research and Innovation, North York General Hospital, Toronto, ON, Canada; Department of Pharmacy, North York General Hospital, Toronto, ON, Canada; Department of Pharmacy, North York General Hospital, Toronto, ON, Canada; Department of Pharmacy, North York General Hospital, Toronto, ON, Canada; Department of Pharmacy, North York General Hospital, Toronto, ON, Canada; Centre for Research and Innovation, North York General Hospital, Toronto, ON, Canada; Institute of Health Policy, Management and Evaluation, University of Toronto, Toronto, ON, Canada

**Keywords:** chemotherapy, gravimetric, patient safety, sterile compounding, technology, pharmaceutical

## Abstract

**Purpose:**

Interventions to improve the safety and efficiency of manual sterile compounding are needed. This study evaluated the impact of a technology-assisted workflow system (TAWS) on sterile compounding safety (checks, traceability, and error detection), and efficiency (task time).

**Methods:**

Observations were conducted in an oncology pharmacy transitioning from a manual to a TAWS process for sterile compounding. Process maps were generated to compare manual and TAWS checks and traceability. The numbers and types of errors detected were collected, and task times were observed directly or via TAWS data logs.

**Results:**

Analysis of safety outcomes showed that, depending on preparation type, 3 to 4 product checks occurred in the manual process, compared to 6 to 10 checks with TAWS use. TAWS checks (barcoding and gravimetric verification) produced better traceability (documentation). The rate of incorrect-drug errors decreased with technology-assisted compounding (from 0.4% [5 of 1,350 preparations] with the manual process to 0% [0 of 1,565 preparations] with TAWS use; *P* < 0.02). The TAWS increased detection of (1) errors in the amount of drug withdrawn from vials (manual vs TAWS, 0.4% [5/1,350] vs 1.2% [18/1565]; *P* < 0.02), and (2) errors in the amount of drug injected into the final container (manual vs TAWS, 0% [0/1,236] vs 0.9% [11/1,272]; *P* < 0.002). With regard to efficiency outcomes, TAWS use increased the mean mixing time (manual vs TAWS, 275 seconds vs 355 seconds; *P* < 0.001), had no significant impact on average visual checking time (manual vs TAWS, 21.4 seconds vs 21.6 seconds), and decreased average physical checking time (manual vs TAWS, 58.6 seconds vs 50.9 seconds; *P* < 0.001).

**Conclusion:**

In comparison to manual sterile compounding, use of the TAWS improved safety through more frequent and rigorous checks, improved traceability (via superior documentation), and enhanced error detection. Results related to efficiency were mixed.

**Key Points**

- The implementation of a technology-assisted workflow system (TAWS) in a sterile compounding outpatient pharmacy increased the frequency and rigor of safety checks for each compounded preparation.
- The TAWS safety features detected and resolved more errors than the previously used manual compounding process and outperformed the traceability of manual processes by recording photographs and generating time-stamped digital audit logs.
- The use of the TAWS increased preparation time for pharmacy technicians in the cleanroom but decreased the review time for pharmacists.

Manual sterile compounding of chemotherapy is prone to errors, which have caused patient harm and death.^1-4^ This is because manual (ie, nonautomated) processes are dependent on human vigilance; technicians may perform compounding steps from memory rather than following step-by-step instructions, which can lead to variability in compounding practices (eg, preparation of more than 1 admixture at a time^4^), and forgetting or mixing up steps. For example, analysis of manual workflows has shown 11 latent errors, 3 of which are catastrophic and depend solely on human vigilance to prevent.^5^ Furthermore, manual compounding relies on human double checks, which are vulnerable to interruptions, miscommunication, and delays (eg, waiting for a team member to become available).^5-7^ Human fallibility also remains a risk factor that can lead to errors (eg, misreading drug labels or syringe measurements or unnecessary costs (eg, failing to use a used partial vial before it expires^8^). For example, research shows humans have misread syringes (by at least 1 syringe graduation) 4% of the time,^9^ and adding a double check does not reliably detect accuracy.^10^ Manual compounding has been associated with dose deviation errors of greater than 10% in up to 23% of preparations^11,12^; overdosing may harm patients^13,14^ and underdosing may impair the effectiveness of treatment.^15^ Manual compounding also requires additional effort from staff to sign off on or document compounding steps, which can slow compounding throughput, result in incorrect or illegible documentation, and be difficult to search (eg, paper records cannot be easily searched). Interventions are urgently needed to improve chemotherapy compounding safety and efficiency.

Novel technologies with sensitive error detection techniques, including barcode verification and digital photograph reviews,^16-20^ gravimetric workflow systems,^21-24^ and robotic compounding^25-27^ have been developed to address manual compounding risks. These technology-assisted workflow system (TAWS) capabilities are purported to reduce reliance on human memory by providing standardized workflow prompts, improve measurement accuracy,^9,11,12,28^ create a time-stamped audit trail of compounding steps with photographic records, simplify drug inventory management, and reduce transcription errors.

Despite the claimed benefits of TAWS use, there is a lack of rigorous safety and efficiency data. Studies comparing manual versus TAWS processes have had methodological challenges in collection of both efficiency data (eg, use of unsynchronized punch clocks, which impacted measurement of task time)^29^ and safety data (eg, comparing manual vs TAWS error rates for different medications and reliance on self-reporting,^30^ which may lead to underestimation of errors^31^). Further, multicenter evaluations have reported varying impacts across institutions but have not fully characterized the workflow processes to understand the differences.^32,33^ Therefore, objectively comparing the safety and efficiency of TAWS versus manual compounding is needed to comprehensively understand their respective benefits and drawbacks, thereby informing evidence-based interventions to prevent compounding errors and patient harm from continuing.

To this end, we compared manual and TAWS sterile compounding in a Canadian community oncology ambulatory care pharmacy on (1) safety, as measured by the frequency and type of checks, traceability (documentation of preparation), and error frequency, and (2) efficiency, as measured by compounding task time.

## Methods

### Setting.

This study was conducted at the an oncology pharmacy in Toronto, Canada. The pharmacy operated on weekdays, from 8 am to 4:30 pm, providing treatments to approximately 30 patients per day and compounding approximately 55 preparations per day. The daily operational team consisted of 2 or 3 pharmacists and 2.5 pharmacy technician full-time equivalents (all dedicated to the oncology pharmacy and compounded sterile preparations).

### Study design.

This was an observational study in which we collected data on a common set of drugs before and after they were transitioned from a manual to a TAWS compounding process. Institutional ethics board approval was acquired (REB #20-0026) and pharmacy staff provided consent.

### Intervention: description of TAWS.

The TAWS evaluated was the Pyxis IV Prep (formerly CATO) system, version 2.46.08 (BD, San Diego, CA). The TAWS provides barcode verification of compounding materials, step-by-step compounding instructions, and a combination of a precise weight scale along with a database of densities for different drugs and fluids to calculate the precise dose or volume being manipulated (ie, gravimetric verification). To support documentation of the components and amounts of drug and diluent used, photographs are automatically taken throughout the TAWS workflow. Table 1 contrasts features employed by the TAWS against the manual workflow.

**Table 1.** Description of Error Types and Impact

Error type	Description of error	Impact of error	Workflow differences
Used partial vial errors			
Used partial vial not used when available	Failure to use a used partial vial (ie, drug vial containing leftover medication) that is within its use date	Unnecessary and costly drug waste when vials expire without being used (Note: Although there is no patient safety impact, we classify this as an error for simplicity.)	Manual: relies on mental recall of availability of used partial vial or visual scan of cleanroom to see if a used partial vial is present TAWS: automated prompt that used partial vial is available
Selection errors			
Incorrect container selected	Use of wrong type of container (syringe vs IV bag vs elastomeric infuser) or use of container of incorrect size or material or with incorrect diluent; for example, IV bags vary in size (eg, 250 mL vs 500 mL), material (eg, PVC vs non-PVC plastic), and diluent (eg, 5% dextrose in water vs 0.9% sodium chloride injection)	Could lead to patient receiving drug in wrong concentration, with incompatible diluent, or for wrong duration	Manual: visual inspection by cleanroom technician or pharmacist when reviewing key steps through webcam (ie, video review^a^) TAWS: barcode scan identifies error; detection prior to product’s use prevents errors and subsequent costs of wasted product
Incorrect **drug s**elected	Use of drug vial other than what is prescribed; includes errors in drug concentration or brand	Can cause severe harm or death; can be difficult to detect because most drugs are clear colorless liquids, so injection of incorrect drug is often indistinguishable from injection of correct one	Manual: visual inspection by cleanroom technician or pharmacist when reviewing key steps through webcam (ie, video review^a^) TAWS: barcode scan identifies error; detection prior to product’s use prevents errors and subsequent costs of wasted product. (Note: Different brands of the same drug may not be distinguishable to the TAWS, depending on the institution’s configuration.)
Measurement errors			
Incorrect amount of reconstitution diluent withdrawn	Too little or too much diluent drawn up in preparation to inject into powdered drug	May lead to incorrect concentration of drug and compromise dose accuracy for subsequent compounding steps	Manual: technician relies on mental recall or checks an information sheet for amount to use; followed by visual inspection by cleanroom technician or pharmacist when reviewing key steps through webcam (ie, video review^a^) TAWS: step-by-step instruction of type and amount of diluent to use; gravimetric weight scale verifies correct amount
Incorrect amount of reconstitution diluent injected	Too little or too much diluent injected into powdered drug	Will compromise dose accuracy for all subsequent compounding steps, and the error may affect multiple patients if same drug vial is used to provide doses for different preparations; if underdiluted, powder may not be dissolved completely	Manual: no check possible (injection amount cannot be verified visually once injected) TAWS: gravimetric weight scale verifies correct amount
Incorrect container adjustment amount	Adding or removing wrong amount of diluent in IV bag or elastomeric infuser (no diluent is required for syringe containers) Elastomeric infusors must be filled with the correct amount of diluent, whereas IV bags need to have fluid withdrawn to make room for the volume of drug being injected.	Can alter concentration of compounded product	Manual: no check possible (adjustment amount cannot be verified visually once adjustment performed) TAWS: gravimetric weight scale verifies correct amount (Note: For elastomeric infusers, a cleanroom technician programs automated pump to inject required amount of fluid into container. The technician does not verify the accuracy of the pump for each preparation, but 2 pharmacy team members calibrate the pump at the start of the day.)
Incorrect amount of dose withdrawn	Too little or too much drug is drawn into syringe(s)	Potential patient harm from under- or overdosing	Manual: visual inspection by cleanroom technician or pharmacist when reviewing key steps through webcam (ie, video review^a^) TAWS: gravimetric weight scale verifies correct amount
Incorrect amount of dose injected	Too little or too much drug injected into final container	Will result in dose error and can cause patient harm	Manual: no check possible (injection amount cannot be verified visually once injected) TAWS: gravimetric weight scale verifies correct amount

Abbreviations: IV, intravenous; PVC, polyvinyl chloride; TAWS, technology-assisted workflow system.

^a^The real-time video review is triggered by the cleanroom technician verbally calling out “Check please!”, stating the patient’s name, and then showing the drug label to the video camera, which is being viewed remotely by a pharmacist. The pharmacist then reads the label and identifies the corresponding patient’s order in their own order records. This approach minimizes bias by leaving it to the pharmacist to manually reconcile the drug name, dosage, container, and quantities of drug or reconstitution diluent present in the syringe being presented to the video camera.

### Data collection procedure.

The observers recorded task time and errors detected by pharmacy staff or TAWS (details are provided below); observers did not identify errors themselves. Three human factors specialists familiarized themselves with the compounding workflow via in-person observations and orientation by pharmacy staff during the month prior to data collection. Observers also collected pilot data to develop the data collection form in Microsoft Excel for Mac (Microsoft Corporation, Redmond, WA) and generated a process map, which included all checks and documentation. During formal data collection, 2 observers were present on each observation day and conducted a daily debriefing and merging of observations data sets in the late afternoon.

#### Timeline.

Formal data collection occurred over 4 months (August 17-December 11, 2020) in 3 phases: preimplementation (August 17-September 14), acclimation (September 15-October 30), and postimplementation (November 1-December 11). Data on manual preparations were collected in all 3 phases because medications were transitioned to TAWS workflow gradually. In the preimplementation phase, data collection occurred only for manual preparations. On September 15, the pharmacy team transitioned 11 medications to TAWS workflow. During the acclimation phase, no task time data was collected since staff were still adjusting, potentially impacting their efficiency. However, TAWS safety features (eg, barcode scanning, gravimetric verification) were immediately active, so error data from the acclimation phase was included. Starting on November 1 (the start of the postimplementation phase), both safety and task time data from TAWS preparations were included for analysis. See eAppendix Table A1 for additional detail.

#### Sample size.

Pharmacy records from the prior year showed roughly 600 preparations were performed per month for the 11 medications being transitioned to TAWS compounding. As in a prior study by other researchers,^23^ we planned to collect data on 600 manual and 600 TAWS preparations. However, in mid-study the pharmacy leadership changed plans and transitioned all medications to TAWS compounding. Therefore, we included data on all compounded medications, and the sample size exceeded 1,000 preparations in both the manual workflow and TAWS data. (Note: the pharmacy transitioned medications to TAWS compounding in a rolling fashion. Therefore, observers collected data on manual preparations in parallel with gathering of data on TAWS preparations until all medications had been transitioned to TAWS compounding [November 25, 2020]. Regardless of when medications were transitioned to TAWS preparation, task time was recorded as of November 1, 2020.)

#### Inclusion and exclusion criteria. 

We included all medications for which a patient label was printed. However, we excluded oral medications, batch-compounded medications (ie, doses not specific to a patient), and medications prepared less than 7 times in either workflow. Additional exclusions specific to each analysis are described below.

### Safety metrics.

The evaluated safety metrics consisted of checks and traceability, errors, and dose accuracy (for the TAWS preparations only, since there is no comparable method of accurately assessing dose deviation in the manual workflow).

#### Checks and traceability.

Process maps of manual and TAWS workflows were generated based on the observations and analyzed to quantify and characterize the types of checks and traceability procedures (ie, documentation) produced by both workflows.

#### Errors.

Observers recorded the following errors: (1) “within-date” unused partial vials (UPVs) not used, (2) incorrect container selected, (3) incorrect drug selected, (4) incorrect amount of reconstitution diluent withdrawn, (5) incorrect amount of reconstitution diluent injected, (6) incorrect container adjustment, (7) incorrect amount of dose withdrawn, and (8) incorrect amount of dose injected. Table 1 provides descriptions of each error type. In the manual compounding process, errors were detected by pharmacy staff. Specifically, observers recorded when pharmacy staff verbalized a concern to another team member and requested a change (eg, an incorrect drug was provided to the cleanroom technician, who asked for a corrected vial). The observers did not attempt to detect any errors besides those detected and verbalized by the pharmacy team. Similarly, errors in TAWS process were detected by verbalizations by pharmacy staff. However, errors were also detected by reviewing the data logs for barcode scanning failures or for gravimetric measurements that were flagged as “out of tolerance.” Out-of-tolerance events in TAWS logs were manually reviewed to exclude workflow artifacts (eg, a technician forgot to remove an item from the scale but quickly recognized and resolved the problem). Out-of-tolerance events for dose withdrawals were only counted if they occurred during the final withdrawal required for the ordered dose (the TAWS accounts for earlier errors by adjusting the amount requested in the final withdrawal so errors in early withdrawals do not impact the patient).

#### Dose accuracy.

Dose accuracy cannot be determined in the manual workflow because it depends on a human visually inspecting the amount of drug in a syringe; this visual inspection can only provide a pass-or-fail assessment (ie, it cannot assess how far the measurement is from the ordered dose). In contrast, preparations in TAWS workflow benefit from gravimetric verification checks that can detect minute differences from the ordered dose. Therefore, we only analyzed dose accuracy for TAWS preparations by reviewing the data logs to assess the distribution of dose measure deviations. We calculated “percent dose deviation” by subtracting the ordered dose from the actual dose in the container and dividing that value by the ordered dose. Ordered doses with volumes less than 2 mL (assuming a drug density of 1 g/mL) were excluded from the analysis because the institution configured the TAWS to extend dose tolerances in these cases.

### Efficiency metrics.

Observers recorded task times in Excel using a preformatted worksheet with multiple columns to represent each task substep; observers used keyboard shortcuts to note the time for each substep (eg, the moment a preparation was placed into or taken out of the biologic safety cabinet [BSC]). Duration data collection was found to be reliable between observers when interrater reliability was calculated from pilot observations (intraclass correlation coefficient [ICC] of >0.75^34^). The sample sizes reported for the 3 task times vary because we excluded preparations with incomplete data (eg, because the view of the cleanroom was momentarily blocked).

We calculated 3 task times:

1. Active mix time (the duration from the first item being placed in the BSC to the last item being removed). Interruptions (eg, personal conversations that paused compounding activities) were subtracted from the mix time.
2. Digital check time (the duration of the pharmacist’s video or photo review in the manual and TAWS workflow, respectively). In the TAWS process, duration was collected from the TAWS data logs. While the data logs included all preparations made with the TAWS, we only included data from preparations the observers saw in person.
3. Physical check time (the duration of the pharmacist physically handling and inspecting the final preparation to placing it in a bin for transport). Interruptions were subtracted to ensure unrelated distractions (eg, personal conversations) did not influence comparisons between workflows.

### Data analysis.

All statistical tests were conducted on IBM SPSS Statistics for Macintosh, Version 27.0 (IBM Corporation, Armonk, NY). Differences in error frequency between the manual and TAWS processes were analyzed using a chi-square test, and differences in task time were evaluated using a *t* test. Outliers in task duration were excluded. Descriptive statistics of dose accuracy in TAWS gravimetric preparations were calculated in Microsoft Excel.

## Results

Five out of 5 pharmacists (100%) and 11 out of 12 technicians (92%) were observed. Interrater reliability among 3 observers was established by comparing 202 observations. This produced an average ICC of 0.96 (minimum, 0.87; maximum, 0.999).

### Safety metrics.

***Checks and traceability*.** Table 2 presents comparative data on the differences in verification checks and traceability for the manual and TAWS workflows across 7 compounding steps. The exact numbers of checks varied by preparation, but the manual workflow included 3 or 4 verification checks, whereas the TAWS workflow included 6 to 10 checks. More checks were conducted for preparations requiring reconstitution (eg, checking that the correct amount of diluent was used) or a container (eg, checking that the correct type and size of intravenous bag or elastomeric infuser was used). For select medications, the TAWS in our institution was not configured to require gravimetric measurements (the specific density of the drug is not known, so gravimetric calculations cannot be performed). These volumetric preparations therefore require 2 to 4 fewer checks than gravimetric preparations.

**Table 2.** Comparison of Checks and Traceability in Manual and TAWS Workflows Per Preparation

Compounding step	Manual workflow			TAWS workflow (assuming full gravimetric workflow)		
	Verification check type	No. of checks	Traceability	Verification check type	No. of checks	Traceability
1. Staging (gathering materials to send into cleanroom)	Visual inspection of what was staged by cleanroom technician	1	Lot number of drug vial is recorded on paper form at end of preparation	(1) Barcode scan during staging of materials (outside cleanroom); (2) barcode scanning (inside cleanroom) just prior to use of each component^a^; (3) visual inspection by cleanroom technician	3	Automatic time-stamped electronic records only created for barcode scanning that occurs inside cleanroom; records include failed or accepted barcode scans, as well as drug vial lot numbers
2. Measuring diluent for reconstitution	Real-time video review by pharmacist (requested by cleanroom technicians)	1	No records produced	Gravimetric verification via weight scale	1	(1) Automatic time-stamped electronic documentation of volume drawn up; (2) photographic record of syringe with diluent amount
3. Confirming injection of diluent for reconstitution	No verification check	0	No records produced	Gravimetric verification via weight scale	1	Automatic time-stamped electronic documentation of volume injected
4. Adjusting container volume^b^	No verification check	0	No records produced	Gravimetric verification via weight scale	1	Automatic time-stamped electronic documentation of volume adjusted
5. Measuring drug for injection	Real-time video review by pharmacist (requested by clean room technicians)	1	Paper form initialled by pharmacist	Gravimetric verification via weight scale	1	(1) Automatic time-stamped electronic documentation of dose drawn up; (2) photographic record of syringe with drug amount
6. Confirming injection of drug into container	No verification check	0	No records produced	Gravimetric verification via weight scale	1	(1) Automatic time-stamped electronic documentation of dose injected; (2) photographic record of final container and drug vial(s) used
7. Releasing labelled product to nursing area	In-person visual inspection by pharmacist	1	(1) Paper form initialled by pharmacist to indicate final inspection done; (2) paper form initialled by clean room technician for accountability; (3) initials of cleanroom technician and pharmacist recorded digitally in Microsoft Access^c^	(1) Photographic inspection by pharmacist; (2) in-person visual inspection by pharmacist	2	Electronic record of approval of photographs; paper form is initialled by pharmacist after final product is inspected; remaining dose and barcode of used partial vial also recorded to aid in future preparations that may use the used partial vial
Summary	The manual workflow has a range of 3-4 checks, depending on the workflow. Documentation is paper based, with cleanroom technicians and pharmacists making extra effort to manually sign off on paper-based forms, which are later stored and require manual human review if preparation details are needed. There is no record of what the pharmacists approve through the video camera. Technicians rely on reference documents to know which diluent to use and the appropriate quantity to use (or memorized these details).			The TAWS workflow has a range of 6-10 checks, depending on the workflow. Documentation is electronic, with cleanroom technicians’ and pharmacists’ actions automatically tracked by the electronic system. Photographs of key steps in the preparation, as well as gravimetric measurements, are captured as part of the preparation record, and the digital signatures of the accountable technician and pharmacist are captured automatically. The TAWS workflow standardizes the sequence of compounding steps across technicians given the automated workflow. The step-by-step procedure minimizes the need for memorization (eg, which diluent to use for which medication, when to perform each step), which simplifies training.		

Abbreviation: TAWS, technology-assisted workflow system.

^a^Barcode scanning of all components does not occur up front. Components are verified “just in time” prior to use to reduce risk of selection errors.

^b^Container adjustments involve removal of fluid from an intravenous bag prior to injection of the prescribed drug, or the addition of a diluent to an elastomeric infuser prior to injection of prescribed drug.

^c^Microsoft Access is a registered trademark of Microsoft Corporation, Redmond, WA.

There are important differences in the traceability produced by the 2 workflows. In the manual workflow, pharmacy staff are required to manually sign off on and record information on paper forms to indicate their involvement in preparation, and record key details (eg, the lot numbers of the drug vials used). In the TAWS workflow, documentation of each verification check (eg, barcode scan result, gravimetric measurement, photographs of preparation, staff involved) is automatically and electronically recorded in data logs.

A visual depiction of the process changes encountered when converting a manual workflow to a TAWS workflow is captured in the eAppendix (Figure A1).

#### Errors: UPV not used.

There were 2 incidents where a within-date UPV was not used when available (one in the manual compounding process and one in the TAWS process). In the manual preparation process, a vial of albumin-bound paclitaxel was thrown out in error, but the error was detected by a cleanroom technician, who remembered that they had used the vial earlier in the day. During TAWS preparation a paclitaxel vial was thrown out in error, but the availability of the UPV was flagged by the TAWS and resolved. As a result, there were no significant differences between the 2 processes with regard to this metric.

#### Errors: drug and container selection.

The rate of errors involving incorrect drug selection decreased significantly with TAWS use (manual vs TAWS, 0.4% [5/1,350] vs 0% [0/1,565]; *P* < 0.02), but no significant difference was found for container selection errors (manual vs TAWS, 0.2% [3/1,350] vs 0% [0/1,565]). Four of the drug selection errors were due to mix-ups of trastuzumab products, specifically Herceptin (Genentech) and Ogivri (Mylan Institutional Inc.). Table 3 summarizes these data, and further details on each selection error are described in the eAppendix (Table A2).

**Table 3.** Comparison of Selection Errors in Manual and TAWS Workflows

Error type	Workflow and sample size	Errors detected, No. (%)	Description	Statistical significance
Incorrect drug selected	Manual (N = 1,350)	5 (0.37)	Incorrect drug detected by cleanroom technicians (n = 3) and pharmacists during video review (n = 2)	χ ^2^(1; 2,915) = 5.81; *P* < 0.02
	TAWS^a^ (N = 1,565)	0 (0)	NA	
Incorrect container selected	Manual (N = 1,350)	3 (0.24)	Incorrect IV bag type detected by cleanroom technician (n = 1), pharmacists in video review (n = 1), and physical check (n = 1)	Not significant
	TAWS^a^ (N = 1,565)	0 (0)	NA	

Abbreviations: IV, intravenous; NA, not applicable; TAWS, technology-assisted workflow system.

^a^As described in Table 2, the TAWS workflow includes 2 barcode scans. Selection errors detected and resolved via the first barcode scan are not recorded; only errors in the second scan are recorded. As a result, the true detection rate is likely underestimated.

#### Errors: measurement.

Table 4 presents the number of detected measurement errors in the manual versus the TAWS workflow. In the TAWS workflow, there was a significantly higher rate of detection of measurement errors during withdrawal of a drug from a vial (manual vs TAWS, 0.4% [5/1,350 vs 1.2% [18/1,565]; *P* < 0.02) and during injection of a drug into the final container (manual vs TAWS, 0% [0/1,236] vs 0.9% [11/1,272]; *P* < 0.002). No significant differences were found in the frequency of reconstitution errors (manual vs TAWS, 0/148 vs 0/250) or container adjustment errors detected (manual vs TAWS, 0.1% [1/1,236] vs 0.3% [4/1,272]). Table 4 also shows that error deviations varied considerably. Deviations were calculated as follows: ([volume weighed – volume expected]/volume expected) × 100. Container adjustment errors deviated by –16.9% to 98.5%, dose withdrawals errors from –87.7% to 14.1%, and dose injection errors from –14.7% to –5.2%. These data show that the TAWS detected and prevented several concentration or dosing errors. Of note, the sample sizes for each error type vary because some errors only occurred for certain preparation types (eg, preparations for which reconstitution is not always required and those administered as syringes could not encounter container adjustment errors). Further details on each detected error are provided in the eAppendix (Tables A3-A6).

**Table 4.** Comparison of Measurement Errors Detected in Manual and TAWS Workflows

Error type	Workflow and sample size	Errors detected, No. (%)	Description of error	Statistical significance	Error deviation by volume (TAWS only)
Incorrect amount of reconstitution diluent withdrawn	Manual (N = 148)	0 (0)	NA	NA	Not measurable
	TAWS (N = 250)	0 (0)	NA	NA	NA
Incorrect amount of reconstitution diluent injected	Manual (N = 148)	NA	Not detectable without TAWS	NA	Not measurable
	TAWS (N = 250)	0 (0)	NA	NA	NA
Incorrect container adjustment amount	Manual (N = 1,236)	1 (0.1)	Overwithdrawal from IV bag	Not significant	Not measurable
	TAWS (N = 1,272)	4 (0.3)	2 errors due to over- or underwithdrawal from IV bag; 2 errors due to underinjection into elastomeric infuser	Not significant	–16.9% to 98.5%
Incorrect amount of dose withdrawn	Manual (N = 1,350)	5 (0.4)	2 errors due to under-withdrawal; 2 errors due to overwithdrawal; for 1 error, cause not determined	χ ^2^(1,2915) = 5.63; *P* < 0.02	Not measurable
	TAWS (N = 1,565)	18 (1.2)	9 errors due to underwithdrawal; 9 errors due to overwithdrawal.		–87.7% to 14.1%
Incorrect amount of dose injected	Manual (N = 1,236)	0 (0)	Errors not detectable without TAWS	χ ^2^(1; 2,508) = 10.74; *P* < 0.002	Not measurable
	TAWS (N = 1,272)	11 (0.9)	All 11 errors were underinjections; 3 preparations had ordered doses of exactly 2 mL		–14.7% to –5.2%

Abbreviations: IV, intravenous; TAWS, technology-assisted workflow system

#### Dose accuracy of TAWS preparations.

The average dose deviation of the final compounded product was –0.27% (mean, –0.21%; SD, 1.2%). All preparations made with the TAWS had a dose deviation less than ±5%, and 1,484 out of 2,211 preparations (67%) were within 1% of the ordered dose. Notably, the sample size for the dose accuracy analysis (n = 2,211) was different from that for other TAWS analyses (n = 1,565) because we included all recorded gravimetric preparations even if observers were not present at the time.

### Efficiency metric.

Use of the TAWS increased average mixing time (manual vs TAWS, 275 seconds vs 355 seconds; *P* < 0.001), had no significant impact on visual checking time (manual vs TAWS, 21.4 seconds vs 21.6 seconds), and decreased physical checking time (manual vs TAWS, 58.6 seconds vs 50.9 seconds; *P* < 0.001). Table 5 provides a summary of these findings.

**Table 5.** Comparison of Manual and TAWS Compounding Task Times

Task	Workflow and sample size	Task time, mean (SD), s	Difference in mean task time with use of TAWS, s	Statistical significance
Active mixing (by cleanroom technician)	Manual (N = 1,111)	275 (140.4)		
	TAWS (N = 983)	355 (151.6)	79.7	*t*_2,092_ = 12.5; *P* < 0.001
Digital check (by pharmacist)	Manual (N = 1,128)	21.4 (13.2)		
	TAWS (N = 1,068)	21.6 (12.7)	0.3	Not significant
Physical check (by pharmacist)	Manual (N = 1,202)	58.6 (29.9)		
	TAWS (N = 1,121)	50.9 (25.3)	–7.8	*t*_2,321_ = 6.7; *P* < 0.001

## Discussion

Our findings demonstrated that the TAWS workflow, compared to a manual workflow, offered improved safety and mixed effects on efficiency (ie, reduced time to physical inspection of the final product but increased mixing time in the cleanroom).

Our process mapping revealed that the TAWS workflow involved a higher frequency of technology-supported checks that were more rigorous than human checks in the manual workflow. The increased checks in the TAWS workflow led to significantly increased detection of measurement errors but lower detection of incorrect drug errors. This second finding is misleading due to 2 factors. First, in the manual workflow, incorrect drug errors were detected by the cleanroom technician and verbalized to the staging technician and thus easily noticed and recorded by the observers. In the TAWS workflow, the staging technician was alerted to the error by a barcode scanning failure, and there was no verbalization of the error, so it was missed by observers. Second, the TAWS provides two barcode scanning checks: one for the staging technician and one for the cleanroom technician. The TAWS only records scanning failures from the second scan. That is, the TAWS logs show no incorrect drug selection errors, even if some were detected and resolved by the staging technician at the first scan. Therefore, the absence of drug selection errors in TAWS data logs is likely an indication of its success. This interpretation is supported by previous studies that have shown that TAWS reduced the frequency of selection errors or failures to use UPV vials.^22,35^ Notably, the configuration of TAWS in our institution was not able to detect errors in drug brand (eg, different brands of bortezomib), so further refinements to the TAWS may increase the safety benefits further.

Additionally, the use of gravimetric checks in the TAWS workflow detected significantly more measurement errors than the manual compounding process. This is because manual verification of syringe amounts is vulnerable to human fallibility,^9^ unlike gravimetric measurement supported by a TAWS.^10^ The gravimetric checks ensured a low average dose deviation (–0.27%, n = 2,214) for TAWS preparations in our study, which is in line with previous studies reporting average dose deviations of –0.62% (n = 11,874)^22^ and –0.8% (n = 3,156).^23^ Unfortunately, we were unable to independently assess dose accuracy in the manual workflow (this would have been possible only if we had duplicated key steps in the manual workflow with gravimetric measurements). Therefore, we cannot conclusively state that dose accuracy was improved with TAWS use versus the manual workflow, but TAWS implementation considerably improved the ability to detect measurement errors.

Measurement errors were detected in 2.1% of our TAWS preparations, which was lower than the 7% to 8% detection rate reported in other studies evaluating TAWS compounding.^22,24,27^ Variation in error rates, as well as changes over time, has been documented in multicenter studies evaluating TAWS use.^24,27^ Future studies exploring the impact of contextual factors (eg, staffing ratios, workflow configuration) on error rates may help inform efforts to optimize safety.

While the safety benefits of TAWS use are unequivocal, its impact on efficiency is more nuanced. In our study the TAWS process increased mixing time in the cleanroom but reduced the task time for physical checking activities; this finding had precedent in a prior study.^23^ The increased mixing time with a TAWS workflow versus a manual workflow is due to the increased checks (Table 2). These additional checks generate a richer audit trail automatically, whereas the manual workflow requires technicians to sign off on the preparation on a separate paper form and also write the drug vial lot number used. A further benefit of a TAWS workflow was its replacement of the video checks that were required in the manual workflow. Video reviews required cleanroom technicians to interrupt pharmacists to ask for them to visually inspect and approve the amounts measured in a syringe through the camera feed. This simultaneously delayed cleanroom technicians and raised safety issues for pharmacists, who had to interrupt their review of drug orders for other patients. Therefore, the increased mixing time required by the TAWS offset the streamlining of the review process, as pharmacists could review the photographs at a time of their choosing and also retain the photographs as part of a preparation’s documentation.

The automatic electronic documentation that a TAWS produces may also generate other efficiencies. For example, TAWS tracking of drug inventory (including UPVs) may eventually replace inventory and restocking tracking activities currently handled by the staging technician in between preparations; this was outside the scope of our study. We also did not compare the time required to train staff on the manual process versus the TAWS process. Given that a TAWS process provides considerable decision support and reduces the need for memorization, it may reduce training time and decrease barriers to increasing the number of staff available to compound. Additional research on the cost implications of these time savings would be helpful to provide a broader analysis of the impact of a TAWS on pharmacy efficiency.

We also found that pharmacists required less time to physically inspect the final compounded product in the TAWS process versus the manual process despite the processes being identical (although TAWS preparations had an additional label to review). One possible reason for the time reduction is that photographic review in the TAWS process occurs at a time of the pharmacist’s choice, unlike in a manual workflow wherein pharmacists are interrupted to perform a video review. As a result, pharmacists may be able to better coordinate the timing of the photographic review with the physical check, thereby increasing the efficiency of the latter. In our study the switch to a photographic review may also have improved the safety and efficiency of pharmacists’ review of new clinical orders, as they were no longer interrupted to verify compounding accuracy via video checks; this aspect of the pharmacists’ workflow was outside the scope of our study and not measured, but it may be a useful metric to record in future studies.

### Limitations.

There were several limitations to our study. First, there are benefits of TAWS use that were not explicitly captured in our findings. For example, during manual compounding, pharmacists were regularly interrupted to perform a video double-check of key steps, which may have impacted the efficiency and accuracy of those interrupted tasks (eg, drug order verification and transcription tasks) but which we did not collect data on. Second, our sample size may have been insufficient to evaluate infrequent errors (eg, incorrect-container errors). Third, the human visual verification of measured doses in the manual workflow was not accurate enough to assess dose deviation errors. As a result, we were unable to compare dose accuracy between the manual and TAWS workflows. Fourth, observers did not independently check preparations for errors that the pharmacy team missed; it is possible more errors occurred in the manual workflow than we have reported here. Fifth, we used a short timeframe for the pharmacy staff to acclimate to the new system; the task times we reported may have been different if we had delayed TAWS data collection. Sixth, we did not compare the difference in *total* preparation time between the manual and TAWS processes because the 2 processes ran in parallel during the study. For example, multiple manual and TAWS preparations were staged together or were deliberately shuffled depending on which medication a patient needed first; this could have artificially altered the overall processing time for some preparations for reasons that were unrelated to the workflow used. As a result, our efficiency analysis compared task times for compounding steps that were directly comparable (eg, steps performed by a pharmacy staff member working on a single preparation at a time).

## Conclusion

Our study found that a TAWS improves sterile compounding safety. Use of a TAWS increased the detection of measurement errors and resulted in fewer drug selection errors (eg, drug mix-ups). Further, the TAWS automatically documented all verification checks (including photographs) to provide a complete record of the preparation, whereas the manual workflow required manual, paper documentation. While these added safety benefits come with an increased mixing time in the cleanroom, many hospitals may find the trade-off worthwhile.

## Supplementary Material

zxac167_suppl_Supplementary_Appendix
