# Supplementary material for: Impact of technology-assisted versus manual sterile compounding on safety and efficiency in a Canadian community hospital
Source: Am J Health Syst Pharm. 2022 Jun 15;79(19):1685–96. doi: 10.1093/ajhp/zxac167 (PMC9494253; doi:10.1093/ajhp/zxac167)
Supplement: zxac167_suppl_Supplementary_Appendix [file zxac167_suppl_supplementary_appendix.docx]

# eAppendix—Supplementary material

Workflow Diagram Outlining Changes from Manual to Technology Assisted Workflow 2

Further Details on Sample Size in Study Analyses 4

Variability in the Sample Size of Fluorouracil (5-FU) 4

Exclusions from Dose Accuracy Analyses 4

Details of Selection Errors (Both Manual and TAWS) 7

Details of Manual Measurement Errors 9

Details of TAWS Measurement Errors 10

Errors for Container Adjustments out of Tolerance 10

Errors for Drug Withdrawals Out of Tolerance 11

Errors for Drug Injections Out of Tolerance 13

# Workflow Diagram Outlining Changes from Manual to Technology Assisted Workflow

Figure A1 provides a detailed step-by-step description of the compounding process.

Figure A1. The XXX oncology compounding workflow (simplified) is depicted with the original workflow steps in blue, and new TAWS process additions in orange (assumes gravimetric workflow; volumetric preparations do not have gravimetric checks and rely on pharmacists reviewing photographs of all syringe measurement steps). Manual workflow steps that were eliminated by the implementation of TAWS are crossed out. The points at which detection of selection or measurement errors occur are highlighted in red for both workflows (manual error detection points on the left, TAWS error detection points on the right). Error detection facilitated by the TAWS have a bold border.


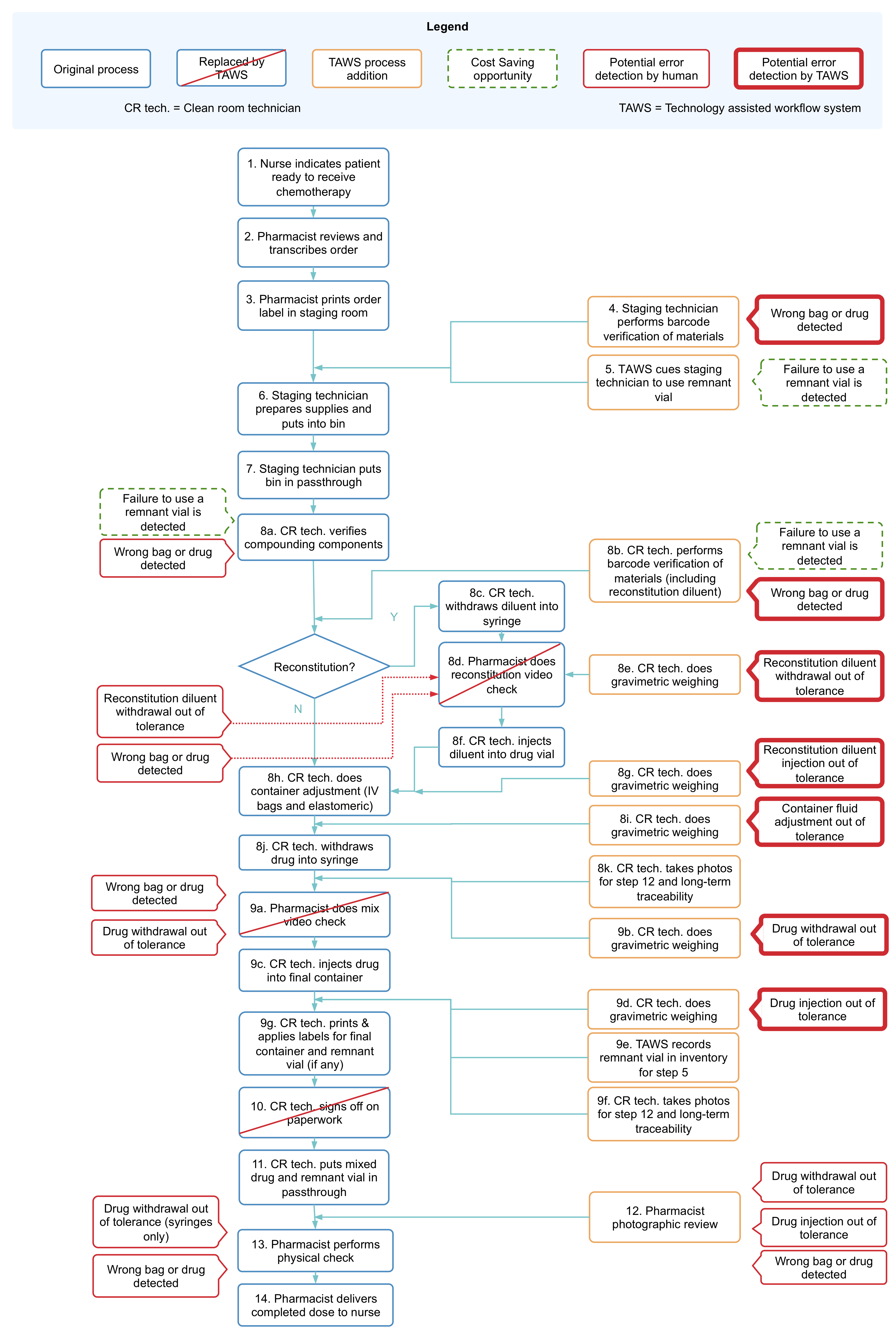


# Further Details on Sample Size in Study Analyses

The sample sizes used in the analyses vary for a number of reasons. A more detailed table is included in Table A1 (the three timeframes are described in the main text of the article).

## Variability in the Sample Size of Fluorouracil (5-FU)

Compounding of 5-FU is prescribed to a patient in three formats at our institution: a) 5-FU syringe alone, b) 5-FU elastomeric infusor alone, c) 5-FU in both syringe and infusor formats (e.g., patient requires a loading dose and then a long duration ambulatory infusion).

When both a syringe and infusor are required, the staging technician combines the materials for both into a single bin, and they are compounded together by the cleanroom technician. As a result, the observers recorded these two preparations for the patient as a single mix (e.g., compounding time). However, there are analyses where they need to be treated separately (e.g., dose accuracy). This means that sample sizes for 5-FU may vary between analyses.

## Exclusions from Dose Accuracy Analyses

TAWS preparations that were not observed by the observational team were still included for analysis of dose accuracy. However, we excluded all preparations that were not gravimetric (e.g., volumetric preparations, manually confirmed, non-specific batch compounding) because they would not capture dose variability.

We also excluded any preparations with an ordered dose less than 2 grams (based on a dose volume of 2mL and assuming a density of 1g/mL) because the institution configured the TAWS to extend allowable dose deviations for these small doses. These small doses are rare in our adult oncology pharmacy.

Table A1. Explanation of how Manual to TAWS Mixes Analyses were performed (CATEGORY 1 ANALYSES)

| Type of Analysis | Summary of analysis and source of data | Timeframes Included | | | Timeframe Rationale | Outliers | Other Inclusions or exclusions |
| --- | --- | --- | --- | --- | --- | --- | --- |
|  |  | **TF** | **Manual** | **TAWS** |  |  |  |
| Dose Accuracy | The TAWS data logs capture the percent dose deviation of the final product (i.e., (actual – ordered dose)/ (ordered dose)*100) of the final product. This is then averaged across mixes to estimate dose accuracy. | 1 | ✖ | ✖ | TAWS logs in TF 1 are excluded because TAWS was still being configured at the time.  Dose accuracy was not estimated for manual preparations because there is no gravimetric data to use. | We are specifically looking for dose outliers, so no outliers were excluded | We only included mixes categorized as gravimetric in the TAWS logs. Manually confirmed, batch preparations or volumetric preparations were excluded.  Dose deviation of reconstitution diluent, and diluent injection were not used to analyze manual accuracy because TAWS adjusts for the concentration of reconstituted drug, and if drug withdrawal is correct, technicians will in most cases fully inject into the final container.  In addition, mixes with a final product less than 2 grams were excluded from the analysis because the institution configured the system to extend tolerances in these cases. |
|  |  | 2 | ✖ | ✔ | Timeframes 2 and 3 are included because any gravimetric TAWS mixes performed after the go-live date should have the benefits of higher dose accuracy based on the gravimetric weighing system.  Dose accuracy was not estimated for manual preparations because there is no gravimetric data to use. |  |  |
|  |  | 3 | ✖ | ✔ |  |  |  |
| Errors | This analysis compares the count of discrepancies between manual and TAWS mixes (e.g., wrong drug or bag staged, wrong dose compounded).  Observers counted the number of times a discrepancy was detected by the clinical team.  The TAWS data logs were also manually reviewed to count discrepancies that were detected by the TAWS system. This includes dose deviations out of tolerance (e.g., too much or too little drug withdrawn). | 1 | ✔ | ✖ | Manual safeguards are present throughout all three time frames, so all 3 timeframes of manual mixes are included.  TAWS was not fully configured and staff had not been trained on the use of TAWS in timeframe 1. Therefore only TAWS mixes from timeframe 2 and 3 were included in the error analysis, because the safety benefits of TAWS would only be fully realized at these times. | Qualitative analysis – no outliers possible | We included gravimetric and volumetric preparations in our error analysis of the TAWS logs. Manually confirmed or batch preparations were excluded.  In addition, TAWS logs contain records of mixes that were not directly observed by the research team (e.g., observers were on lunch break or entering data at end of day). As a result, the manual review of TAWS data logs was restricted to preparations that were observed by the research team (in timeframes 2 and 3 only as described to the left). All other mixes recorded in the TAWS system were excluded. |
|  |  | 2 | ✔ | ✔ |  |  |  |
|  |  | 3 | ✔ | ✔ |  |  |  |
| Duration per mix (active mix time, pharmacist digital and physical check) | Observers captured the start and end of pharmacy technicians’ and pharmacists’ activities, including interruptions to their tasks. See “Metrics” in the body of the article.  Interruptions to staff were subtracted from all times except for the TAWS photographic review, which was calculated solely from the TAWS software logs (i.e., observers were not able to monitor the pharmacists workflow for interruptions during the photographic review.). Note that moments where pharmacy technicians were ‘waiting for the pharmacist’ to perform the video review was not considered an interruption and remained part of the mix time. | 1 | ✔ | ✖ | Manual mix times from all 3 timesframes were included.  Only TAWS mixes from timeframe 3 are included in this analysis. This is because TAWS was still being configured in timeframe 1, and technicians were still acclimating to TAWS in timeframe 2. | Outliers were excluded. | The following mixes were excluded:   - start or end timestamp missing - any drugs that were only mixed with manual, or TAWS - any drugs that were mixed less than 7 times in either manual or TAWS |
|  |  | 2 | ✔ | ✖ |  |  |  |
|  |  | 3 | ✔ | ✔ |  |  |  |

# Details of Selection Errors (Both Manual and TAWS)

In Table A2 below, we describe the selection errors from both manual and TAWS workflows. The barcode scanning features of TAWs prevented selection errors from occurring, and therefore there were almost no errors to detect with TAWS (a single used partial vial error was detected in the TAWS workflow). In contrast, the pharmacy team detected 3 “incorrect container selected” errors, 5 “incorrect drug selected” errors, and 1 used partial vial (UPV) error in the manual workflow.

While there is no distinctive pattern to the reasons behind the incorrect container being selected, we noted patterns in the other error categories. First, the incorrect drug was often selected due to mix-ups of Ogivri and Herceptin. These are two brands of trastuzumab, which is a medication frequently compounded in the pharmacy. These mix-ups are likely due to staging technicians simply falling into a routine and staging the unintended brand for use, despite the brand name being being indicated on the label (e.g., “trastuzumab inj (HERCEPTIN)”). Second, both UPV errors occurred due to the accidental disposal of a vial with usable drug quantity remaining. This is likely due to the fact that the rapid visual inspection of multiple empty vials may result in an occasional misperception of the vial. Glass vials with low drug quantities remaining may appear empty due to parallax effect, and pharmacists working under high workload an time pressure may have mistakenly disposed of the vials when this occurred.

Table A2. Details of Each Selection Error (manual and TAWS processes)

| Workflow | Index | Drug | Detection method | Description |
| --- | --- | --- | --- | --- |
| Incorrect Container Selected | | | | |
| Manual | b158 | Etoposide | Clean room technician | Should have staged a bag of non-PVC normal saline but sent a bag of dextrose instead. |
| Manual | a345 | Cisplatin | Physical check | Details of the discrepancy (wrong material, fluid, or volume) unknown. |
| Manual | p268 | Rituximab IV | Video check | Should have staged a bag of NS 500 ml, unknown what was actually staged. |
| Incorrect Drug Selected | | | | |
| Manual | b595 | Trastuzumab | Clean room technician | Should have staged Ogivri instead of Herceptin. |
| Manual | b661 | Trastuzumab | Video check | Should have staged Herceptin instead of Ogivri. |
| Manual | b807 | Trastuzumab | Video check | Should have staged Ogivri instead of Herceptin. |
| Manual | b918 | Trastuzumab | Clean room technician | Should have staged Ogivri instead of Herceptin. |
| Manual | b974 | Bevacizumab | Clean room technician | Details unknown, but wrong vials were staged, meaning it was a mix-up between Avastin and MVASI. |
| Used Partial Vial not used when Available | | | | |
| Manual | b948 | Abraxane | Clean room technician | The used partial vial (UPV) from an earlier mix was accidentally thrown out by the pharmacist during physical check. Clean room technician remembered that a UPV existed, informed pharmacists, and found it in the waste bin. |
| TAWS | p431 | Paclitaxel | Staging technician | The UPV from an earlier mix was accidentally thrown out by the pharmacist during physical check. Because the UPV was recorded in TAWS, the staging technician knew to look for it in the waste bin. |

# Details of Manual Measurement Errors

In Table A3 below, we describe 6 measurement errors from the manual workflow; 1 error was due to a container fluid adjustment, detected during real-time video review by the pharmacist, and 5 were incorrect dose measurements. We did not detect a pattern or clear underlying cause with regards to these errors; 2 doses were too low, 2 were too high, and we were unable to ascertain what the discrepancy was for the 5^th^ error. The real-time video review was the primary mechanism for detecting the measurement was incorrect, and in one case a pharmacist noted the dose was incorrect during the final inspection because the dose was prepared in a syringe and therefore the volume was easily readable.

Table A3. Details of Measurement Errors (manual processes only)

| Discrepancy | Index | Drug | Detection method | Description |
| --- | --- | --- | --- | --- |
| Container fluid adjustment | b244 | Paclitaxel | Video check | Pharmacy ran out of stock of 500 ml non-PVC normal saline bags, so they staged a larger size. Clean room technician needed to withdraw enough fluid to bring the volume to 500 ml. During video check, the pharmacist thought the clean room technician had withdrawn too much, and asked them to re-inject some. |
| Wrong dose withdrawn | a208 | Rituximab IV | Video check | Pharmacist noticed during video check that the clean room technician withdrew too little drug by 2 ml. Instructed clean room technician to withdraw more drug. |
| Wrong dose withdrawn | a644 | Rituximab IV | Video check | Pharmacist noticed during video check that the clean room technician withdrew too much drug. Instructed clean room technician to push some back into vial. |
| Wrong dose withdrawn | p5 | Bevacizumab | Video check | Pharmacist noticed during video check that the clean room technician withdrew too much drug. Instructed clean room technician to push some back into vial. |
| Wrong dose withdrawn | p70 | Bevacizumab | Clean room technician | Clean room technician held up the withdrawn drug syringe for video check, but adjusted the volume in the syringe realizing a change was needed. This means there was either too much or too little withdrawn. |
| Wrong dose withdrawn | b687 | Bortezomib | Physical check | Pharmacist noticed during physical check that the completed syringe was short by 0.5 ml. Sent back in to clean room for technician to fix. |

# Details of TAWS Measurement Errors

## Errors for Container Adjustments out of Tolerance

In Table A4 below, we describe 4 measurement errors when adjusting the container volume from the TAWS workflow; 2 were associated with an intravenous (IV) bag, and 2 were associated with an elastomeric infusor. The errors with the IV bags involved 1 case of withdrawing too little fluid, and another with withdrawing too much. In contrast, the elastomeric infusors both involved an under-injection of fluid. Regardless of the type of container, the TAWS weight scale was a helpful safety feature capable of verifying the diluent volume adjustment was accurate.

Table A4. Details of Container Adjustment Out of Tolerance Events (TAWS only)

| # | Index # | Drug Name | Vehicle | Adjustment (inject vs withdraw fluid) | Technician’s Action (mL) | What TAWS wanted (mL) | Deviation volume (mL) | Final adjustment after error message (mL) |
| --- | --- | --- | --- | --- | --- | --- | --- | --- |
| 1 | a258 | pamidronate | Elastomeric | Inject | +379 | +390 | -10.57 | +383 |
| 3 | p559 | etoposide | IV bag | Withdraw | -21.8 | -11 | 10.84 | -11.133 |
| 5 | p1345 | 5-FU elastomeric | Elastomeric | Inject | +179 | +193 | -13.37 | +191.99 |
| 6 | p1375 | gemcitabine | IV bag | Withdraw | -53.96 | -65 | -11.03 | -59.45 |

## Errors for Drug Withdrawals Out of Tolerance

In Table A5 below, we describe 18 measurement errors recorded in the TAWS workflow, specifically when measuring the final withdrawal requested by TAWS. Please note, the TAWS request is not necessarily the prescribed dose. For example, if the prescribed dose equates to 100mL, and the cleanroom technician has drawn up 90mL from drug vials and needs to measure a final 10mL to complete the prescribed dose, the measurement error described here refers to the error made for the final 10mL measurement. This is because errors in this final withdrawal is most closely correlated to errors in the final doses. Errors in withdrawals earlier in the process are inconsequential because TAWS will simply compensate for the volume needed up to the final withdrawal.

The dose deviations captured for this final withdrawal stage ranged from an under-dose of 87.7% to an overdose of 14.1%. We have highlighted 7 errors where TAWS was requesting 2 mL or less to “complete” the prescribed dose on the final withdrawal. This is a relatively small amount of medication in absolute terms and is more difficult for clean room technicians to perform. However, all 7 of these errors were for doses with a volume of less than 5mL, so even small errors were a large percentage deviation from the prescribed dose; therefore the demand for accuracy is warranted. Interestingly, in 2 of the 7 small volume errors (Table A5 rows #6 and #14), had the technician not corrected the error, the final dose would still have been within 5% of the prescribed dose. This suggests in rare instances, TAWS may demand a higher accuracy than is necessary.

The largest errors in absolute terms are found on rows 13 and 18, where the cleanroom technician withdrew 5mL too little. This suggests that errors larger than 5mL are likely to be captured by human visual inspection and corrected prior to performing weight measurements with TAWS.

We detected no clear pattern in the types of errors committed in Table A5.

Table A5. Details of last dose withdrawal out of tolerance events (TAWS only)

| # | Index # | Drug Name | Ordered dose (mg) | Volume of ordered dose (mL) | What Technician Withdrew (mL) | What TAWS wanted (mL) | Deviation volume (mL) | Dose Deviation for the withdrawal (%) | Dose Deviation of Final Product if Withdrawal error not caught and perfect injection |
| --- | --- | --- | --- | --- | --- | --- | --- | --- | --- |
| 1 | a68 | Bortezomib | 2.7 | 2.7 | 0.109 | 0.899 | 0.79 | -87.77 | -96.02% |
| 2 | a132 | 5-FU elastomeric | 4150 | 83.0 | 59.728 | 63.978 | 4.25 | -6.64 | -5.12% |
| 3 | a198 | Bortezomib | 2.3 | 2.3 | 0.169 | 0.739 | 0.57 | -77.04 | -92.68% |
| 4 | p292 | Leucovorin | 630 | 63.0 | 24.816 | 22.634 | 3.18 | 14.06 | 5.05% |
| 5 | p517 | Doxorubicin | 17 | 8.5 | 9.000 | 8.5 | 0.5 | 5.88 | 5.88% |
| 6 | p877 | EPOCH (etoposide) | 90 | 4.5 | 1.274 | 1.086 | 0.19 | 17.38 | **0.3%** |
| 7 | p901 | Trastuzumab | 378 | 17.2 | 9.644 | 8.559 | 1.08 | 12.68 | 6.18% |
| 8 | p909 | Bortezomib | 2.3 | 2.3 | 1.164 | 1.294 | 0.13 | 12.74 | -49.80% |
| 9 | p930 | EPOCH (etoposide) | 90 | 4.5 | 4.689 | 4.5 | 0.19 | 4.19 | 4.19% |
| 10 | p1262 | Carboplatin | 220 | 22.0 | 22.895 | 22.0 | 0.9 | 4.07 | 4.07% |
| 11 | p1268 | Bortezomib | 2.1 | 2.1 | 2.269 | 2.1 | 0.12 | 5.37 | 5.37% |
| 12 | p1250 | Fludarabine | 65 | 2.6 | 1.144 | 1.484 | 0.34 | -23.02 | -13.15% |
| 13 | p1342 | Pertuzumab | 420 | 14.0 | 6.618 | 11.698 | 5.08 | -43.42 | -36.3% |
| 14 | p1413 | Trastuzumab | 88.2 | 4.0 | 1.440 | 1.267 | 0.17 | 13.63 | 4.14% |
| 15 | p1415 | Docetaxel | 142 | 14.2 | 9.549 | 14.2 | 4.65 | -32.75 | -67.25% |
| 16 | p1530 | Vincristine | 2 | 2.0 | 1.807 | 1.997 | 0.19 | -9.66 | -9.66% |
| 17 | p1624 | Bleomycin | 13.2 | 4.4 | 3.403 | 4.343 | 0.94 | -21.57 | -21.57% |
| 18 | p1630 | Leucovorin | 455 | 45.5 | 13.581 | 18.631 | 5.05 | -27.09 | -11.09% |

## Errors for Drug Injections Out of Tolerance

In Table A6 below, we describe 11 measurement errors recorded in the TAWS workflow, specifically when injecting the prescribed dose into an IV bag or elastomeric infusor. In all 11 errors, the clean room technician injected less than what was requested by TAWS. This is expected because it is difficult to squeeze every last drop of medication out of a syringe. In addition, the closed transfer system contains dead volume which cannot be expelled. The deviation from desired injection volume ranged from -14.7% to -5.16% (the threshold for an error is 5%). Ten out of the 11 errors were due to injections into an IV bag rather than an elastomeric infusor. The largest error in absolute terms was an under-injection of 4.61 mL (row #1), suggesting that injection errors of 5mL or more are more easily detected by human visual inspection.

We have highlighted that 3 of the errors (row 2, 5, and 6) were for 2mL, and therefore challenging to clean room technicians to inject without losing part of the dose to syringe dead volume. In these edge cases, TAWS provides a valuable verification check to ensure that clean room technicians are able to ensure that the prescribed dose is attained in the final container. We did not detect a discernable pattern in the remaining errors, other than that pembrolizumab represented over a third of the errors detected (although technicians were only off by less than 1mL in each case); it is unclear why this is the case. Future research may be required to assess why injection errors were more common with IV bags compared to elastomeric infusors.

Table A6. Details of dose injection out of tolerance events (TAWS only)

| # | Index # | Drug Name | Ordered dose (mg) | Volume of ordered dose (mL) | What Technician Injected (mL) | What TAWS wanted (mL) | Deviation volume (mL) | Dose Deviation on the injection (%) | Final Dose Deviation of Container After Correction (%) |
| --- | --- | --- | --- | --- | --- | --- | --- | --- | --- |
| 1 | a202 | 5-FU elastomeric | 4225 | 84.5 | 80.097 | 84.707 | 4.61 | -5.21% | -0.30% |
| 2 | a318 | vincristine | 2 | 2.0 | 1.865 | 1.955 | 0.09 | -6.76% | -1.45% |
| 3 | a585 | paclitaxel | 123 | 20.5 | 19.367 | 20.597 | 1.23 | -5.53% | -0.19% |
| 4 | a652 | gemcitabine | 1520 | 38.0 | 34.636 | 38.026 | 3.39 | -8.85% | -0.23% |
| 5 | p25 | vincristine | 2 | 2.0 | 1.894 | 1.924 | 0.03 | -5.31% | 0.97% |
| 6 | p153 | vincristine | 2 | 2.0 | 1.855 | 1.975 | 0.12 | -7.25% | 0.97% |
| 7 | p165 | trastuzumab | 451.5 | 20.5 | 18.23 | 21.43 | 3.2 | -14.69% | -0.56% |
| 8 | p975 | pembrolizumab | 115 | 4.6 | 4.359 | 4.709 | 0.35 | -5.23% | 0.04% |
| 9 | p1170 | pembrolizumab | 148.75 | 6.0 | 5.563 | 5.933 | 0.37 | -6.50% | -0.30% |
| 10 | p1189 | pembrolizumab | 200 | 8.0 | 6.971 | 7.961 | 0.99 | -12.86% | -1.46% |
| 11 | p1485 | pembrolizumab | 172.5 | 6.9 | 6.544 | 6.664 | 0.12 | -5.16% | -4.74% |
